# Effects of Optokinetic Stimulation on Verticality Perception Are Much Larger for Vision-Based Paradigms Than for Vision-Independent Paradigms

**DOI:** 10.3389/fneur.2018.00323

**Published:** 2018-05-09

**Authors:** Katja M. Dockheer, Christopher J. Bockisch, Alexander A. Tarnutzer

**Affiliations:** ^1^Department of Neurology, University Hospital Zurich, Zurich, Switzerland; ^2^Department of Otorhinolaryngology, University Hospital Zurich, Zurich, Switzerland; ^3^Department of Ophthalmology, University Hospital Zurich, Zurich, Switzerland; ^4^University of Zurich, Zurich, Switzerland

**Keywords:** subjective visual vertical, subjective haptic vertical, graviception, otolith, optokinetic nystagmus

## Abstract

**Introduction:**

Verticality perception as assessed by the subjective visual vertical (SVV) is significantly biased by a rotating optokinetic stimulus. The underlying mechanisms of this effect remain open. Potentially, the optokinetic stimulus induces a shift of the internal estimate of the direction of gravity. This hypothesis predicts a shift of perceived vertical using other, non-vision dependent, paradigms as well. Alternatively, an optokinetic stimulus may only induce a shift of visual orientation, and so would be task specific.

**Methods:**

To test this prediction, both vision-dependent SVV and vision-independent [subjective haptic vertical (SHV)] paradigms were applied. In 12 healthy human subjects, perceived vertical was measured in different whole-body roll positions (up to ±120°, steps = 30°) while watching a clockwise or counterclockwise rotating optokinetic stimulus. For comparison, baseline trials were collected in darkness. A generalized linear model was applied for statistical analysis.

**Results:**

A significant main effect for optokinetic stimulation was noted both for the SVV paradigm (*p* < 0.001) and the SHV paradigm (*p* = 0.013). However, while pairwise comparisons demonstrated significant optokinetic-induced shifts (*p* ≤ 0.035) compared to baseline in all roll-tilted orientations except 30° and 60° left-ear-down position and counterclockwise optokinetic stimulation for the SVV paradigm, significant shifts were found in only 1 of the 18 test conditions (120° left-ear-down roll orientation, counterclockwise optokinetic stimulation) for the SHV paradigm. Compared to the SHV, the SVV showed significantly (*p* < 0.001) larger shifts of perceived vertical when presenting a clockwise (15.3 ± 16.0° vs. 1.1 ± 5.2°, mean ± 1 SD) or counterclockwise (−12.6 ± 7.7° vs. −2.6 ± 5.4°) rotating optokinetic stimulus.

**Conclusion:**

Comparing the effect of optokinetic stimulation on verticality perception in both vision-dependent and vision-independent paradigms, we demonstrated distinct patterns. While significant large and roll-angle dependent shifts were noted for the SVV, offsets were minor and reached significance only in one test condition for the SHV. These results suggest that optokinetic stimulation predominately affects vision-related mechanisms, possibly due to induced torsional eye displacements, and that any shifts of the internal estimate of the direction of gravity are relatively minor.

## Introduction

Accurate and precise internal estimates of the direction of gravity are essential for spatial orientation, navigation, and postural stability. This is achieved by integrating input from various sensory systems, including the vestibular organs (i.e., the semicircular canals and the otolith organs), vision, and proprioception (1). The otolith organs—composed of the utriculus and the sacculus—are head-based sensors that directly measure the gravito-inertial force vector, reflecting the sum of gravitational force and inertial force due to linear acceleration (2–6). Other sensory input as provided by joint and muscle receptors and skin pressure sensors is trunk-based and is, therefore, represented in a distinct reference frame. According to Bayesian observer theory, the brain integrates all available sensory cues in a weighted fashion according to their relative reliabilities and prior likelihood in order to generate an internal estimate of the direction of gravity (6–10).

Experimentally, internal estimates of the direction of gravity can be quantified both at the level of brainstem reflexes [for example, by measuring ocular counter roll (11)] and perceptually at the level of higher cortical functions. A popular means to assess verticality estimates is to require subjects to adjust a luminous line or arrow along the perceived direction of gravity [for review, see Ref. (12)]. Whereas for this, task adjustments will be very accurate near the upright position, systematic errors in verticality perception occur when roll-tilted: while for small roll angles (up to approximately 60°), variable shifts of perceived vertical away from the side of roll-tilt have been reported [representing roll over-compensation, termed “E-effect” (13)], offsets toward the side of roll-tilt [i.e., roll under-compensation, termed the “A-effect” (14)] up to 40° can be observed for larger roll angles, peaking around 120–135° of whole body roll-tilt (9, 15). Besides estimates being less accurate when roll-tilted, repeated measurements of perceived vertical also demonstrated a roll-angle-dependent modulation of trial-to-trial variability. Specifically, with increasing whole-body roll-tilt, the precision (i.e., the degree of reproducibility) of adjustments decreases (9, 15–17), reaching a minimum around 120–135° of roll-tilt and showing intermediate values in the upside-down position (6). Simulations using a Bayesian optimal observer model suggested that this roll-angle-dependent modulation of subjective visual vertical (SVV) precision is both due to the electromechanical properties of the otolith sensors (i.e., their non-uniform distribution of preferred stimulation directions and their non-linear firing rate) and central computation mechanisms that are not optimally tuned for roll-angles distant from upright (6).

Specific psychophysical paradigms designed for measuring perceived vertical differ in the sensory signals used and may, therefore, show distinct response patterns. While the most popular approach, the SVV, integrates visual input, other paradigms such as the subjective haptic vertical (SHV) or the subjective postural vertical (SPV) do not (13–15, 18–20). By eliminating visual input, adjustment errors when roll-tilted were significantly reduced, supporting the notion that deviations between perceived and actual earth vertical arise from central processing of visual information (20).

Previous studies in healthy human subjects have shown that a rotating optokinetic stimulus is a powerful means to introduce a systematic bias in verticality perception, shifting the SVV significantly toward the direction of rotation (21–24). Most recently, Ward and colleagues have addressed the role of dynamic visual stimuli on the SVV while roll-titled (25). They predicted a stronger weight on extra-vestibular (e.g., visual or proprioceptive) sensory input signals with increasing whole-body roll angle. This assumption was based on the observation that the reliability of otolith sensory input for generating the internal estimate of the direction of gravity decreases with increasing roll angle (6). Measuring the amount of bias introduced by the optokinetic stimulus, they found a significant roll-angle dependency, with shifts growing with roll angle. Thus, visual input was weighted more when vestibular input became less reliable, confirming their hypothesis and supporting earlier findings from Dichgans and colleagues (21). At the same time, the precision remained unaffected by the dynamic visual surround, further emphasizing the leading role of otolith input for precise estimates of the direction of gravity.

The underlying mechanisms of this optokinetic-induced bias of the SVV, however, are not fully understood. Potentially, the optokinetic stimulus adds an offset to the internal estimate of the direction of gravity. This hypothesis predicts a shift of perceived vertical using other, vision-independent paradigms as well. Alternatively, the optokinetic stimulus may only induce a shift of visual orientation, e.g., by a torsional displacement of the eyes, and so would be task-specific. This alternative hypothesis predicts an unbiased internal estimate of the direction of gravity when using an experimental paradigm that does not use visual orientation cues.

## Materials and Methods

### Subjects

This study was carried out in accordance with the recommendations of the Cantonal Ethics Committee Zurich with written informed consent from all subjects. All subjects gave written informed consent in accordance with the Declaration of Helsinki. The protocol was approved by the Cantonal Ethics Committee Zurich (study protocol 2016-00943). Potential participants were screened for vestibular impairment by use of a standardized questionnaire. Two subjects had to be excluded because of discomfort and claustrophobia while sitting on the turntable. Twelve healthy right-handed human subjects (8 females and 4 males; aged 20–55 years; mean age ± 1 SD: 31.3 ± 12.3 years) were included in the study. One of them was familiar to the experimental protocol and had participated in a previous study using the same optokinetic stimulus (25), the others were naïve.

### Experimental Setting

**Figure 1:**
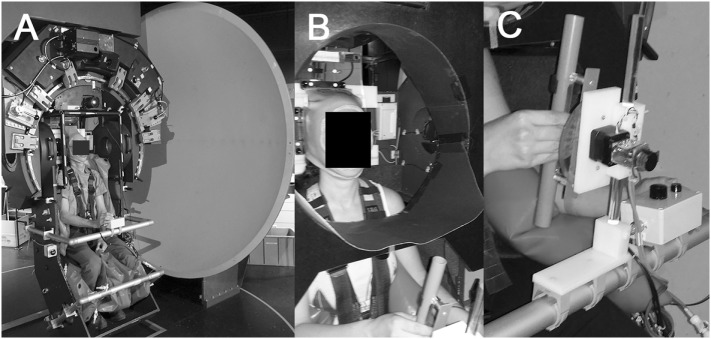
Illustration of the experimental setup used. All trials were collected on a three-axis motorized turntable **(A)**. Details of the thermoplastic mask used to stabilize the subject’s head and to restrict the peripheral visual field downwards (so that the subject could not see the bar during the adjustments) are shown in **(B)**. Subjects grasped the bar with the right hand and confirmed adjustments by pressing a button placed left of the bar **(C)**.

Subjects were seated on a turntable with three servo-controlled motor-driven axes (prototype, built by Aucotronic, Jona, Switzerland; see Figure 1A). For security and stabilization, a four-point safety belt fixating the hips and shoulders, and pillows placed beside the trunk, shoulders and legs were installed. A thermoplastic mask (Sinmed, Reeuwyk, The Netherlands; Figure 1B), individually adapted and fixated on a platform behind the head, kept the head and trunk in alignment. The roll axis of the turntable corresponded to the naso-occipital line passing between the eyes. A sphere in front located at 1.5 m distance to the eyes served as projection surface for a laser-generated arrow (red color; length 500 mm; width 3 mm; covering the central 9.5° of the binocular visual field) and for the optokinetic stimulus, respectively [see Figure 1 from Ref. (25)]. The rotating optokinetic stimulus was composed of randomly distributed white dots on a black background and generated using the Psychophysics Toolbox (26, 27) and GNU Octave (version 3.2.3). Three different trial conditions were applied: baseline (no optokinetic stimulation), a clockwise rotating optokinetic stimulus (optokinetic CW), and a counterclockwise rotating stimulus (optokinetic CCW). Myopic participants wore their glasses. In the vision-based paradigm, subjects were asked to move the arrow and align it with perceived vertical, i.e., along the direction of gravity, using a knob in front of them fixed on a safety bar. In the non-visual-based paradigm, they indicated perceived vertical by adjusting a plastic tube (length 29 cm, diameter 2.5 cm; Figure 1C) that was mounted on a safety bar placed in front in the midline in 40 cm distance. “By adding a Velcro strip to one end of the tube, the two ends could be haptically distinguished. To achieve this haptic task, subjects actively explored the area in front of them in darkness with the right arm being unrestrained to determine the starting roll orientation of the rod. Subjects were allowed to reach the rod in a manner they felt most comfortable. Due to the fixed location of the rod 40 cm in the front of the midline, this will result in a slight forward movement of the unrestrained right arm, flexion in the elbow, and an approximately horizontal position of the forearm for accurate grasping of the rod,” as previously described by Schuler et al. (20). The whole experiment took place in complete darkness, except for the luminous arrow and the optokinetic stimulus. To avoid any visual orientation cues when adjusting the bar in front due to the illumination of the optokinetic stimulus, the caudal parts of the participant’s peripheral visual field were restricted by a circular, wraparound visual cover (Figure 1B), that limited the field of view to about ±36°. Turntable, arrow, and bar orientation signals were digitized at 200 Hz and stored on a computer hard disk for offline processing.

### Experimental Protocol

Perceived vertical was measured in various static whole-body positions in the roll plane (upright, ±30°, ±60°, ±90°, ±120°; random order), either using the SVV or the SHV (two sessions). Altogether, 324 trials in random order were collected [12 trials per condition (*n* = 3; baseline, optokinetic CW, optokinetic CCW) and roll orientation (*n* = 9)] in each subject and session. For turntable roll position movements, constant accelerations and decelerations of ±10°/s^2^ were applied. As these acceleration values were clearly above the detection threshold of semicircular canal stimulation (28) and self-motion perception (29), they could potentially have affected adjustment performance (30, 31). Therefore, SVV and SHV trials started 5 s after the turntable had reached its final position, as after such a delay, post-rotatory torsional ocular drift (i.e., systematic changes in torsional eye position over time) at the time subjects confirm arrow adjustments was found to be small (11). An acoustic signal indicated the start of all trials. Depending on the trial type, the optokinetic stimulus was presented at the same time and depending on the session, the arrow (SVV only) was shown as well. Subjects were instructed to align the arrow or the bar along the shortest path possible with the perceived direction of vertical. While doing so, subjects were asked to look straight ahead. Adjustment time was restricted to 5 s for all trials to control for an accuracy bias, e.g., to avoid spending more time in more difficult (most likely larger) roll orientations (32). Starting orientation of the arrow was random within the entire roll plane, whereas the starting position of the bar was restricted to random CW or CCW offsets of 28–84° relative to the subject’s roll orientation due to the physical constraints of the human wrist. For the SHV, trials started only after subjects had identified and correctly grasped the tactile device placed in front of them in darkness. Before starting measurements, subjects practiced about 10–15 SVV and SHV adjustments until they were able to perform them within the time limit. Both the SVV and the SHV experiments lasted about 110 min and were recorded on different days. A short break in upright position with the lights on was provided in the middle of each session.

### Data Analysis

Data were extracted and sorted according to the whole-body roll orientation and the condition for each subject using interactive programs written in Matlab 2017b (The MathWorks, Natick, MA, USA). According to the right-hand rule, deviations into the CW direction were positive and deviations into CCW direction had a negative sign. Differences in adjustment errors and variability values for baseline trials and test trials (optokinetic CW/CCW) were calculated for both the SVV and SHV paradigm. Furthermore, effects of optokinetic stimulation (“Δ optokinetic”) on accuracy and precision in the two paradigms were compared. As our data were normally distributed (Jarque–Bera hypothesis test of composite normality, jbtest.m, Matlab 2017b), mean ± 1 SD values were provided when pooling individual data points. Statistical analysis was performed using SPSS 24 (IBM, Armonk, New York, NY, USA). We applied a generalized linear model for all statistical analyses if not specified otherwise. Main effects included the trial condition (*n* = 3; optokinetic off vs. optokinetic CW vs. optokinetic CCW), the direction of rotation of the SVV arrow or the bar (*n* = 2; CW vs. CCW), and turntable position (*n* = 9). The level of significance was kept at *p* = 0.05, and Fisher’s least significant difference method was used to correct for multiple comparisons. Principal component analysis (PCA) was used to perform comparisons between two dependent variables [see Ref. (25) for details on PCA]. The coefficient of determination (*R*^2^) was used to measure the goodness of fit. A correlation between two variables was considered significant whenever the 95% confidence-interval (95% CI) of the slope did not include 0.

## Results

The direction of arrow or bar rotation (CW vs. CCW) had no significant (*p* > 0.05) main effect on adjustment errors or trial-to-trial variability in all three trial conditions. Trials with CW and CCW arrow or bar rotations were, therefore, pooled for further analyses.

### Paradigm 1: SVV

#### Arrow Adjustment Errors

**Figure 2:**
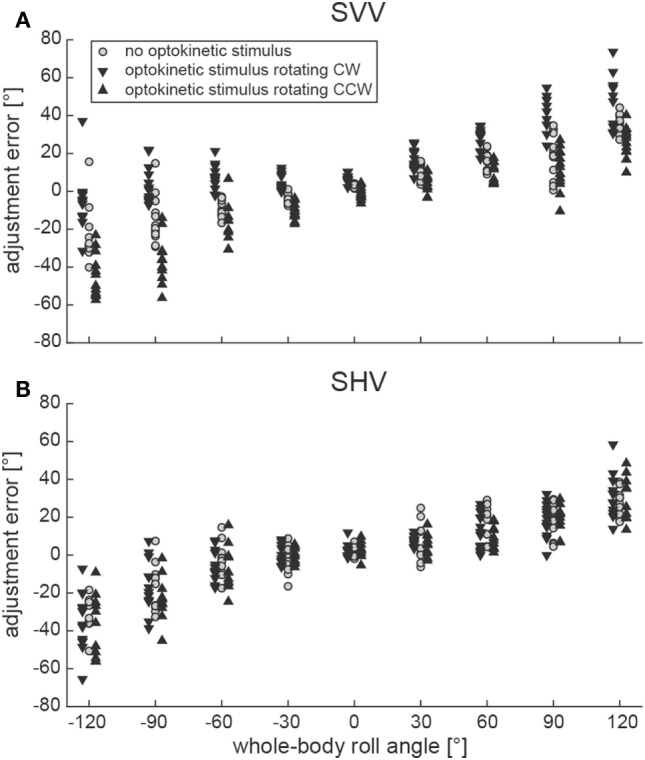
Single adjustments in a representative subject (#10) are plotted against whole-body roll position (roll-tilt up to ±120°, steps of 30°) for both the SVV paradigm **(A)** and the subjective haptic vertical (SHV) paradigm **(B)**. Trials from the three different conditions (gray circles = baseline; inverted black triangles = optokinetic stimulus rotating CW; black triangles = optokinetic stimulus rotating CCW) are shown separately and were slightly displaced laterally for better illustration.

Illustrative data from a single subject (#10) can be found in Figure 2A, indicating a shift of adjustments toward the direction of rotation of the optokinetic stimulus in the test conditions on top of the overall tendency to under-compensate for whole-body roll (as seen also for the baseline trials). Individual adjustment errors for all 12 subjects can be found in the Figure S1 in Supplementary Material.

**Figure 3:**
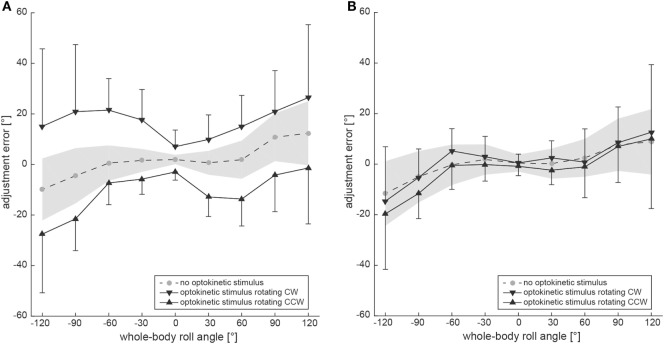
Overall mean adjustment errors for the SVV paradigm **(A)** and the subjective haptic vertical (SHV) paradigm **(B)** are plotted against whole-body roll orientation. Results from the three different trial conditions are shown separately. The gray circles interconnected with a dashed line refer to the baseline SVV measurements (no optokinetic stimulus). The inverted black triangles represent trials with the optokinetic stimulus rotating CW and the black trials refer to trials with the optokinetic stimulus rotating CCW. Whereas black bars reflect ±1 SD for the test trials, a gray shaded-area represents ±1 SD of the baseline trials.

Figure 3A shows group averages, representing the adjustment errors for the three different trial conditions. Whereas SVV adjustments in the baseline condition remained accurate for roll-angles up to ±60° (with an offset of 2.0 ± 1.9° when upright), roll under-compensation was noted for larger roll angles [120° right-ear-down (RED): 12.4 ± 12.8°; 120° left-ear-down (LED): −9.9 ± 12.3°]. For the test conditions, average adjustment errors in upright position were 7.0 ± 6.6° (optokinetic CW) and −3.0 ± 3.3° (optokinetic CCW), reaching average offsets in the range of 15–26° at ±120° roll-tilt for the optokinetic CW condition and average offsets around −1° to −27° at ±120° roll-tilt for the optokinetic CCW condition.

Statistical analysis (generalized linear model) confirmed a main effect for the condition (df = 2, chi-square = 362.04, *p* < 0.001) and the turntable position (df = 8, chi-square = 82.48, *p* < 0.001), while the direction of arrow rotation had no influence (df = 1, chi-square = 0.07, *p* = 0.792). There was a significant interaction between the turntable position and the condition (df = 16, chi-square = 45.49, *p* < 0.001). Pairwise comparisons of adjustment errors for a given roll angle and the different test conditions (optokinetic CW vs. optokinetic CCW) demonstrated significances for all roll-tilted conditions (*p* < 0.001) and for upright position (*p* = 0.021). Pairwise comparisons between the test conditions and the control condition yielded significant differences (*p* ≤ 0.035) for all roll-tilted conditions except at 30° left-ear-down for optokinetic CCW vs. optokinetic off (*p* = 0.085) and at 60° left-ear-down for optokinetic CCW vs. optokinetic off (*p* = 0.056). Differences when upright did not reach significance.

**Figure 4:**
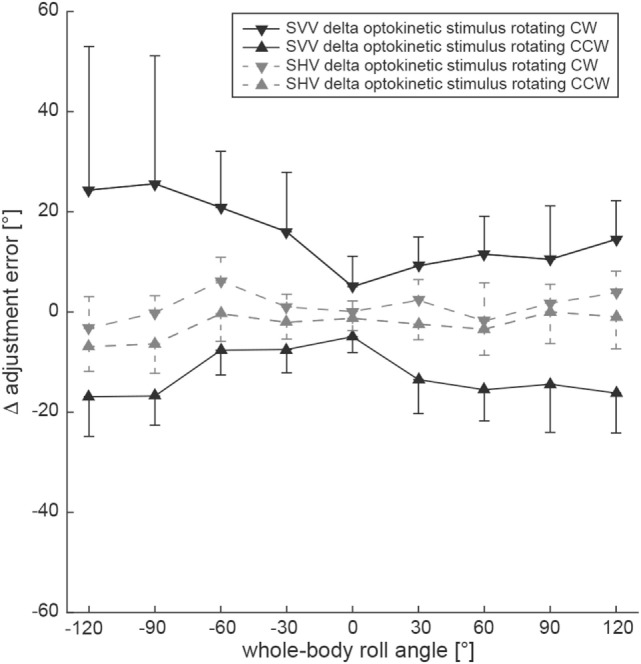
Overall mean individual differences (Δ) in adjustment errors (± 1SD) after subtracting baseline adjustments from the test adjustments for both the SVV (black symbols, interconnected by a solid black line) and the subjective haptic vertical (SHV) (gray symbols, interconnected by a dashed gray line) paradigm are plotted against whole-body roll orientation. Whereas triangles refer to test trials with CCW optokinetic stimulation, inverted triangles refer to test trials with CW optokinetic stimulation.

By subtracting baseline adjustment errors (control condition) from those of the test condition in individual subjects, Δ adjustment errors were calculated (Figure 4). To assess whether the shift induced by either CW or CCW optokinetic stimulation was of comparable size or differed significantly, statistical analysis (generalized linear model) was performed separately using the absolute values of Δ error. Statistical analysis demonstrated a main effect for the two test conditions (df = 1, chi-square = 4.266, *p* = 0.039) and the turntable roll orientation (df = 8, chi-square = 67.06, *p* < 0.001) and a significant interaction between the test conditions and the turntable roll orientations (df = 8, chi-square = 29.22, *p* < 0.001). Pairwise comparisons of Δ adjustment errors for the optokinetic CW and optokinetic CCW conditions indicated that the magnitude (i.e., the absolute values) of the optokinetic stimulation-induced offset for the two test conditions was significantly different (*p* ≤ 0.017) for all left-ear-down roll-tilted positions (with larger offsets for CW optokinetic stimulation), while no significant differences (*p* > 0.05) were noted for all right-ear-down roll-tilted positions.

#### Trial-to-Trial Variability

**Figure 5:**
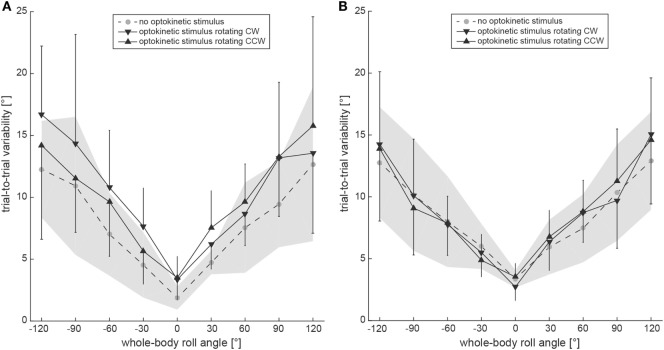
Overall mean trial-to-trial variability values for the subjective visual vertical (SVV) paradigm **(A)** and the subjective haptic vertical (SHV) paradigm **(B)** are plotted against whole-body roll orientation for both test and control trials. Whereas the gray circles interconnected by a dashed line refer to the control condition without optokinetic stimulation, the inverted black triangles indicate variability values from the optokinetic CW condition and the black triangles are linked to the optokinetic CCW condition. The gray-shaded area indicates ±1 SD for the control condition and the bars refer to ±1 SD in the test conditions.

Subjective visual vertical trial-to-trial variability increased with roll orientation in both the test conditions and the control condition (Figure 5A). Statistical analysis (generalized linear model) indicated a main effect for the condition (df = 2, chi-square = 19.43, *p* < 0.001) and for the turntable position (df = 8, chi-square = 267.02, *p* < 0.001). No significant interaction between the conditions and the turntable positions (df = 16, chi-square = 12.22, *p* = 0.729) was found. Pairwise comparisons demonstrated significantly larger variability for both test conditions compared to the control condition (*p* ≤ 0.001), while no significant differences were noted between the two test conditions (*p* = 0.476). For pairwise comparisons at specific roll angles, significant differences between the test conditions and the control were noted at 120° LED (optokinetic CW vs. optokinetic off, *p* = 0.005), at 60° LED (optokinetic CW vs. optokinetic off, *p* = 0.050) and at 30° RED (optokinetic CCW vs. optokinetic off, *p* = 0.049), while no significant differences were observed between the two test conditions.

In a next step, we correlated individual SVV variability values (test conditions) with Δ error (optokinetic CW and CCW pooled) values to evaluate the link between the precision of SVV estimates and the impact of the optokinetic stimulus. Using PCA, we found a significant correlation between these two parameters (*R*^2^ = 0.66, slope = 0.50, 95% CI = 0.35–0.66).

#### Arrow Adjustment Time

On average, SVV trials were completed after 3.0 ± 0.5 s. Statistical analysis revealed no significant main effect for trial duration between the three conditions (df = 2, chi-square = 4.831, *p* = 0.089) or for turntable roll orientation (df = 8, chi-square = 13.67, *p* = 0.091) and no significant interactions. Furthermore, PCA demonstrated no correlation between trial time and absolute adjustment errors (all three conditions pooled) in the SVV paradigm (*R*^2^ = 0.17, slope = 0.03, 95%-CI = 0.03–0.03).

### Paradigm 2: SHV

#### Bar Adjustment Errors

Figure 2B contains raw data from a single subject (#10), indicating no effect of the optokinetic stimulus on adjustment errors in the range of roll-angles tested. Individual adjustment errors for all 12 subjects can be found in the Figure S1 in Supplementary Material. Figure 3B shows pooled data of all subjects, illustrating mean (±1 SD) bar adjustments for both the control condition and the two test conditions. Whereas for baseline adjustments errors were small for upright position (0.4 ± 3.3°) and for roll angles up to ±60°, roll under-compensation was noted for larger roll-angles, reaching values of −11.6 ± 19.6° (120° LED) and 8.8 ± 29.0° (120° RED). A very similar pattern was noted in both test conditions. Again, bar adjustments were accurate for upright (0.5 ± 3.5° and −0.8 ± 3.7°; CW and CCW optokinetic condition, respectively) and small roll-tilt angles (up to ±60°) and demonstrated a tendency for roll under-compensation at larger roll angles (see Figure 3B).

Statistical analysis (generalized linear model) illustrated a main effect for the condition (df = 2, chi-square = 8.634, *p* = 0.013) and the turntable position (df = 8, chi-square = 175.78, *p* < 0.001), while the direction of bar rotation had no influence (df = 1, chi-square = 0.026, *p* = 0.871). There was no significant interaction between the turntable positions and the conditions (df = 16, chi-square = 6.311, *p* = 0.984). With regards to the main effect on the conditions, pairwise comparisons demonstrated significantly different average adjustment errors (overall nine roll orientations) between the two test conditions (optokinetic CW vs. optokinetic CCW, 1.5 ± 15.8° vs. −2.1 ± 16.2°, *p* = 0.006) and between the control condition and the optokinetic CCW test condition (0.5 ± 14.6° vs. −2.1 ± 16.2°, *p* = 0.026). Pairwise comparisons of adjustment errors for a given roll angle and the two test conditions (optokinetic CW vs. optokinetic CCW) demonstrated no significant differences for both upright and roll-tilted positions. Likewise, pairwise comparisons between the test conditions and the control condition yielded no significant differences, except for 120° LED with the optokinetic stimulus rotating CW (*p* = 0.033).

Differences in adjustment errors (Δ error) in the test conditions compared to the control condition are illustrated in Figure 4. Again, to determine whether the shift induced by either CW or CCW optokinetic stimulation was of comparable size or differed significantly, statistical analysis was performed using the absolute values of Δ error. This analysis demonstrated a main effect for the turntable roll orientation (df = 8, chi-square = 113.38, *p* < 0.001) and a significant interaction between the test conditions and the turntable roll orientations (df = 8, chi-square = 16.23, *p* = 0.039), while no main effect for the two test conditions (df = 1, chi-square = 1.793, *p* = 0.181) was noted. Pairwise comparisons of Δ adjustment errors for the optokinetic CW and optokinetic CCW conditions at specific roll orientations indicated that the absolute Δ error values for the two test conditions were significantly different only for 120° LED (*p* = 0.042) and for 90° LED (*p* = 0.005), while for all other roll-orientations, no significant differences were found.

#### Trial-to-Trial Variability

Average trial-to-trial variability in the SHV paradigm was roll-angle dependent for all three conditions, demonstrating increasing variability values with increasing roll angle (see Figure 5B). Statistical analysis indicated a main effect for the turntable position (df = 8, chi-square = 250.85, *p* < 0.001), but not for the condition (df = 2, chi-square = 5.322, *p* = 0.070). No significant interaction between the conditions and the turntable positions (df = 16, chi-square = 20.18, *p* = 0.212) was found. Pairwise comparisons demonstrated a significantly larger variability for the optokinetic CW condition compared to the optokinetic CCW condition (*p* = 0.021), while no significant differences were noted when comparing test and control conditions (*p* > 0.05). For pairwise comparisons at specific roll angles, significant differences between the test conditions and the control were noted at 120° LED (optokinetic CCW vs. optokinetic off, *p* = 0.048), at 60° LED (optokinetic CW vs. optokinetic off, *p* = 0.050), and at 120° RED (optokinetic CW vs. optokinetic off, *p* = 0.012). Between the two test conditions, significant differences in trial-to-trial variability were found for 120° RED (*p* < 0.001). PCA demonstrated no correlation between trial-to-trial variability (test conditions only) and Δ adjustment errors (Δ adjustment errors from trials with CW and CCW optokinetic stimulation pooled) (*R*^2^ = 0.09, slope = 0.93, 95% CI = −0.99 to 1.14).

#### Bar Adjustment Time

On average, bar alignments were confirmed after 2.3 ± 0.6 s. Statistical analysis of trial duration revealed a significant main effect for turntable roll orientation (df = 8, chi-square = 86.18, *p* < 0.001), while no main effect was noted between the three conditions (df = 2, chi-square = 1.676, *p* = 0.432) and no significant interactions were found. With increasing whole-body roll angle, an increase of mean trial duration was noted: whereas in upright position, mean trial duration was 1.9 ± 0.5 s, it reached 2.7 ± 0.7 s at 120° LED. This was also reflected in a significant correlation between the individual trial times (all three conditions pooled) and absolute adjustment errors, with longer trial durations resulting in larger adjustment errors (*R*^2^ = 0.32, slope = 0.05, 95% CI = 0.04–0.06).

### Comparison Between SVV and SHV Adjustments

For comparing the effect size of optokinetic stimulation on both the SVV and the SHV, we used the calculated difference in adjustment error for the baseline condition and the test conditions. We referred to this difference as Δ adjustment errors. Using a generalized linear model, there was a significant main effect for the condition (SVV vs. SHV, df = 1, chi-square = 227.42, *p* < 0.001), but not for the test condition (optokinetic CW vs. optokinetic CCW, df = 1, chi-square = 2.675, *p* = 0.102) and the direction of rotation (df = 1, chi-square = 0.133, *p* = 0.715) (see also Figure 4). Overall, optokinetic-induced shifts in perceived vertical were significantly larger for the SVV than for the SHV. This was true both when presenting a clockwise (15.3 ± 16.0° vs. 1.1 ± 5.2°, all 9 roll orientations pooled) and a counterclockwise (−12.6 ± 7.7° vs. −2.6 ± 5.4°) rotating optokinetic stimulus. Pairwise comparisons for single roll orientations demonstrated significantly (*p* ≤ 0.006) larger shifts for the SVV paradigm compared to the SHV paradigm in all but two positions (upright, 30° RED) when presenting a CW optokinetic stimulus. Likewise, with the optokinetic stimulus rotating CCW, induced shifts were significantly (*p* ≤ 0.009) larger for the SVV paradigm compared to the SHV paradigm in all but three positions (60° LED, 30° LED, upright).

Comparing trial-to-trial variability values, there was no main effect for the paradigm (SVV vs. SHV, df = 1, chi-square = 3.220, *p* = 0.073), while we noted a main effect for the condition (optokinetic off vs. optokinetic CW vs. optokinetic CCW, df = 2, chi-square = 15.07, *p* = 0.001) with pairwise comparisons indicating larger values of the test conditions compared to the control condition. This was true both for CW (*p* < 0.001) and CCW (*p* = 0.002) optokinetic stimulation. In addition, we also noted an interaction between the paradigm and the condition (df = 2, chi-square = 8.947, *p* = 0.011), with pairwise comparisons indicating significantly larger variability for the SVV compared to the SHV when presenting a CW rotating optokinetic stimulus (*p* = 0.006), but not when presenting a CCW rotating stimulus (*p* = 0.090) and in the optokinetic off condition (*p* = 0.184).

## Discussion

Rotating visual orientation cues are a powerful way to bias the SVV (21, 22, 24, 25). However, the underlying mechanisms of these perceptual shifts remain unclear. Potentially, the optokinetic stimulus induces a shift of the internal estimate of direction of gravity as first proposed by Dichgans and co-workers (21). Thus, this hypothesis predicts a shift of perceived vertical using other, vision independent, paradigms as well. When comparing the magnitude of optokinetic bias using either a vision-based (SVV) or a vision-independent paradigm (SHV) in the same study population, however, we noted a striking difference. While significant large and roll-angle dependent shifts were noted for the SVV (with average shifts of 15.3 ± 16.0° and −12.6 ± 7.7° for CW and CCW optokinetic stimuli, respectively), offsets were minor (with average shifts of 1.1 ± 5.2° and −2.6 ± 5.4° for CW and CCW optokinetic stimuli, respectively) and reached significance only in one test condition for the SHV.

Thus, perceived vertical remained accurate in most conditions or showed minor shifts despite an optokinetic rotatory stimulus when using the SHV. This observation suggests that the internal representation of the direction of gravity remains largely unbiased in the presence of an optokinetic rotatory stimulus and, therefore, favors task-specific mechanisms that preferentially modulate visual processing. Similarities and discrepancies in the internal estimate of the direction of gravity when using the SVV and the SHV, respectively, have been previously studied. While current evidence indicates that internal estimates of the direction of gravity for visual and haptic tasks share certain features (20), other aspects, at the same time, seem to be distinct (33).

### Putting Our Results in the Context of Previous Studies

The adjustment errors in the SVV paradigm noted here are similar to previous observations reported by Ward and co-workers, confirming the roll-angle dependent effect of optokinetic stimulation on the accuracy of verticality perception (25). While for the SVV, Δ adjustment errors increased with increasing trial-to-trial variability values, reflecting a roll-angle dependent effect of extra-vestibular (i.e., optokinetic in this case) stimuli on verticality perception (as seen on PCA), this was not the case for the SHV.

Previously, Zupan and Merfeld have studied the effect of a rotatory optokinetic stimulus on the subjective haptic horizontal (SHH) in healthy human subjects while upright (34). They noted two distinct response patterns in their participants. Whereas adjustments remained accurate despite the optokinetic stimulus (rotational velocity = 60°/s) in 12 subjects, five subjects showed marked offsets in the range of 15–20° during prolonged optokinetic stimulation. The reason for this dichotomy of response patterns is unclear. The authors speculated that due to the close distance of the optokinetic stimulus, it was not strong enough to shift perceived vertical in a larger fraction of participants. However, in our experimental setup, we applied a large-field optokinetic stimulus projected to a sphere in 1.5 m distance (covering approximately 70° of the binocular visual field). Nonetheless, shifts of the SHV observed in our study were either minor (and restricted to roll-tilted positions) or (as in most positions) non-significant. Potentially, a build-up effect resulting in circular vection may have contributed to the optokinetic bias in those subjects that presented with significant shifts in the study by Zupan and Merfeld. Since these authors did not provide an analysis of the first 10 s after onset of the optokinetic stimulus, this hypothesis cannot be tested (34). If circular vection contributes to shifts in estimating earth vertical, it should have an impact on verticality estimates independently from the paradigm (SVV or SHV) provided that optokinetic stimulation lasts at least 9 s, which is the average time after onset of an optokinetic stimulus that was needed to perceive circular vection as shown in a study by Thilo and Gresty (35). As in our study, trial time was restricted to 5 s to control for a potential accuracy bias (32), it is unlikely that circular vection played a relevant role here.

Comparing our SHV baseline results with published data sets obtained with the same experimental setup, we noted some discrepancies. We have previously reported hysteresis for the SHV (20) and also a CCW bias of verticality perception for this task when in upright position in complete darkness (36). In our current baseline data, direction of bar rotation did not significantly (*p* > 0.05) affect adjustment errors and average SVH values were accurate when upright or near upright. This might be related to interindividual differences or shorter average trial durations in the current study. Noteworthy, the rotatory optokinetic stimulus had no effect on the trial-to-trial variability in the SHV paradigm, confirming previous observations from the SVV paradigm (25), but contrasting the current SVV paradigm where we noted significantly increased trial-to-trial variability for both test conditions compared to the control condition. This reason for this discrepancy in the SVV paradigm remains unclear.

Furthermore, we noted an increase in average trial duration with increasing whole-body roll angle for the SHV paradigm, but not for the SVV paradigm. This effect was independent from the presence/absence of an optokinetic stimulus and, therefore, could be related to the discomfort of roll-tilted body positions and gravity-related interference with the haptic task.

### Underlying Mechanisms of Optokinetic-Induced Shifts in Verticality Perception

We have previously proposed that it is most likely a combination of effects that leads to the optokinetic-induced bias of verticality perception in the SVV paradigm (25), including an optokinetic nystagmus and torsional deviation of the eyes, a shift in the internal estimate of direction of gravity, and a shift in subjective body roll (24). Conceptually, it is assumed that the brain integrates and weights all available sensory input to generate a unified internal representation of direction of gravity (37). This internal reference is then used for various spatial orientation tasks and combined with task-specific parameters. However, the lack of optokinetic-induced shifts in verticality perception for the SHV paradigm speaks against a bias of the internal representation of direction of gravity. Rather, the optokinetic-induced bias seems paradigm-specific and restricted to vision-dependent paradigms, possibly related to a shift of visual orientation by the optokinetic stimulus.

An optokinetic nystagmus leading to torsional displacement of the eyes due to a rotatory surround represents a potential mechanism (38–42), resulting in offsets when aligning a visual target (43, 44). Such a mechanism, however, requires that the brain is unaware of the torsional position of the eyes, as otherwise a torsional offset will be compensated for. Indeed, previously, this has been suggested to explain systematic roll over-compensation (E-effect) in the subjective visual horizontal for small roll-tilt angles (45). Noteworthy, we did not quantify torsional ocular movements in our study. However, using similar parameters of optokinetic stimulation than applied here [i.e., similar field size (~50°)] and similar rotation velocity [~40°/s, exceeding the saturation level of optokinetic stimulation (46)], a torsional optokinetic nystagmus with a gain of 0.05–0.06 (corresponding to a velocity of 3–3.5°/s) is expected (42). While, therefore, optokinetic-induced torsional displacements of the eyes may provide the basis for the shift in the SVV paradigm, central mechanisms are required as well for modulating the magnitude of the offset as the size of torsional displacement varies little with roll-tilt (some decreases in the amplitude may be expected for larger roll-tilted positions due to static ocular counterroll). Changes in the central weighting of vestibular and extra-vestibular cues for generating internal estimates of direction of gravity have been proposed to explain such roll-angle dependent increases, based on the observation that the reliability of vestibular (otolithic) input decreases with increasing roll angle [see Ref. (25)].

While there are very little data on the effect of optokinetic stimulation on the SHV, several studies have assessed its effect on the postural vertical. Overall, these studies report discrepant findings. When asked to keep the body posture vertical continuously despite lateral tilt disturbances and an optokinetic stimulus, four healthy controls demonstrated shifts in verticality perception that reached a steady state after 17 s with average values of 8.5° (21). However, using verbal reporting of perceived upright during passive roll, Bisdorff and colleagues found no effect of torsional optokinetic stimulation on the SPV (47). Noteworthy, in another study using a similar (passive) paradigm as Bisdorff and colleagues, an effect of rotatory optokinetic stimulation on the SPV was noted (35).

Whereas our SVH-based findings are consistent with those reported from Bisdorff and co-workers (i.e., no effect of optokinetic stimulation on the SPV), they are in disagreement with the observations on the postural vertical from Dichgans and colleagues (21) and Thilo and Gresty (35). While being statistically significant in the latter study (35), the magnitude of these offsets (average shifts of about 1.3° relative to baseline) was very small compared to the effect of optokinetic stimulation on the visual vertical [being in the range of 12–13° for the same rotatory velocity of the optokinetic stimulus (21)].

While differences in the experimental paradigm (passive vs. active adjustments) could explain the discrepancies by Bisdorff and co-workers, the more recent work by Thilo and Gresty speaks against this explanation (as in their study adjustments were passive also). In summary, there is some evidence for a small effect of optokinetic rotatory stimulation on verticality perception in non-visual paradigms with partially conflicting results. This is also reflected in our SHV data with significant, but low amplitude shifts of perceived vertical in a minority of roll orientations tested. However, these effects—although reaching the level of significance—are of much smaller magnitude compared to those observed for vision-based paradigms. Therefore, the conclusion that response patterns to optokinetic stimulation are task-dependent remains valid. The lack of circular vection in our paradigm due to the short trial (and optokinetic stimulus) duration has likely contributed to these differences compared to other studies with longer stimulus duration.

### Limitations

We recorded trial responses within the first 5 s after onset of the optokinetic stimulus, whereas previously published studies on the effect of optokinetic stimuli on verticality perception waited at least 10 s [e.g., Ref. (24, 34)] before trial collection started. While this has the advantage that effects of circular vection in our data sets are minimal, it also limits comparability with previous publications. However, this does not change the general pattern that the magnitude of differences was much smaller compared to the SVV paradigm. At the same time, we cannot exclude that more prolonged (i.e., lasting >10 s) optokinetic stimulation induces more pronounced shifts in perceived vertical also for vision-independent paradigms such as the haptic vertical setup used here.

Furthermore, while shifts in torsional eye position have been shown to affect the percept of vertical in vision-based paradigms but not in non-vision (e.g., haptic) paradigms previously (45), it is likely that optokinetic stimulation-induced eye torsion plays a role in the paradigm-specific effects noted here. To characterize these effects, torsional eye position should be quantified and correlated with behavioral data in future studies.

## Conclusion

While previous research on the mechanisms of optokinetic stimulation-induced biases of verticality perception mainly relied on vision-based paradigms as the SVV, here, we investigated the role of distinct sensory input systems available and emphasized paradigm-specific differences such as stimulus duration and whole-body roll orientation. Comparing the effect of optokinetic stimulation on verticality perception in both vision-dependent and vision-independent paradigms, we demonstrated distinct patterns. While significant large and roll-angle dependent shifts were noted for the SVV, offsets were minor and reached significance only in one test condition for the SHV. These results suggest that optokinetic stimulation predominately effects vision-related mechanisms, possibly due to induced torsional eye displacements, and that any shifts of the internal estimate of the direction of gravity are relatively minor. Discrepancies with previous reports on significant shifts of the SHH or the SPV are likely related to differences in the experimental paradigm and are probably secondary to effects of circular vection. Future experiments, therefore, should further investigate effects of optokinetic stimulus duration and ocular torsion on both vision-dependent and vision-independent paradigms for assessing verticality perception.

## Ethics Statement

This study was carried out in accordance with the recommendations of the Cantonal Ethics Committee Zurich with written informed consent from all subjects. All subjects gave written informed consent in accordance with the Declaration of Helsinki. The protocol was approved by the Cantonal Ethics Committee Zurich (study protocol 2016-00943).

## Author Contributions

KD performed the data collection and drafted the manuscript. CJB helped in the data analysis and interpretation and critically reviewed and edited the manuscript. AAT conceived of the study, participated in the data analysis and the statistical analysis, and critically reviewed and edited the manuscript. All authors read, revised, and approved the final version of the manuscript.

## Conflict of Interest Statement

The authors declare that the research was conducted in the absence of any commercial or financial relationships that could be construed as a potential conflict of interest.
