# Supplementary material for: Effects of Optokinetic Stimulation on Verticality Perception Are Much Larger for Vision-Based Paradigms Than for Vision-Independent Paradigms
Source: Front Neurol. 2018 May 9;9:323. doi: 10.3389/fneur.2018.00323 (PMC5954029; doi:10.3389/fneur.2018.00323)
Supplement: Supplementary file 1 [file Data_Sheet_1.pdf]

Online supplementary material

Supplementary figure 1

Single subject data for both the SVV (left column) and the SHV (right column) paradigm is presented. Mean adjustment errors are plotted against whole-body roll orientation. Results from the three different trial conditions are shown separately. The grey circles interconnected with a dashed line refer to the baseline SVV measurements (no optokinetic stimulus). The inverted black triangles represent trials with the optokinetic stimulus rotating CW and the black trials refer to trials with the optokinetic stimulus rotating CCW. Whereas black bars reflect ±1SD for the test trials, a grey shaded-area represents ±1SD of the baseline trials.

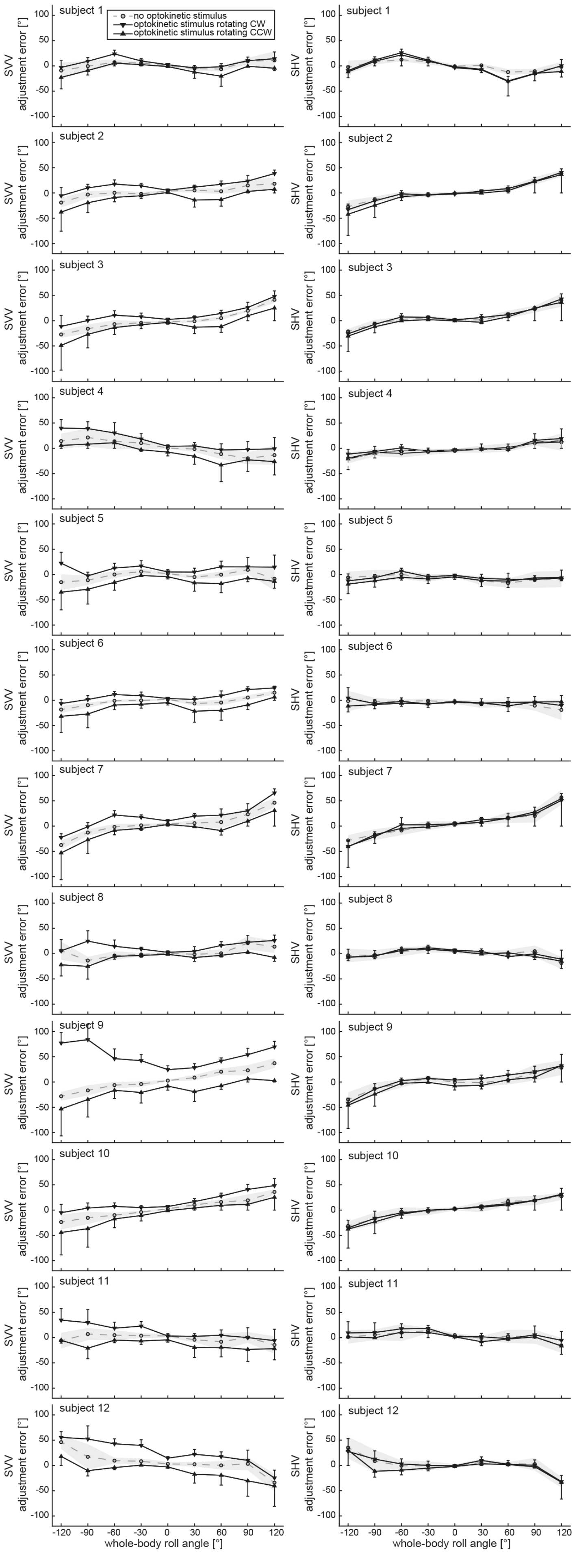
no optokinetic stimulus
optokinetic stimulus rotating CW
optokinetic stimulus rotating CCW
subject 1
subject 2
subject 3
subject 4
subject 5
subject 6
subject 7
subject 8
subject 9
subject 10
subject 11
subject 12
SVV
SHV
adjustment error [°]
100
50
0
-50
-100
-120 -90 -60 -30 0 30 60 90 120
whole-body roll angle [°]
